# Effect of FeCoNiCrCu_0.5_ High-entropy-alloy Substrate on Sn Grain Size in Sn-3.0Ag-0.5Cu Solder

**DOI:** 10.1038/s41598-019-40268-4

**Published:** 2019-03-06

**Authors:** Yu-An Shen, Chun-Ming Lin, Jiahui Li, Siliang He, Hiroshi Nishikawa

**Affiliations:** 1 Joining and Welding Research Institute (JWRI), Osaka University, Ibaraki, 567-0047 Osaka Japan; 2 Department of Mechanical Engineering, Minghsin University of Science and Technology, Hsinchu, 30401 Taiwan; 3 Department of Aviation Mechanical Engineering, China University of Science and Technology, Hsinchu, 312 Taiwan; 4 Department of Electronic Engineering, City University of Hong Kong, Hong Kong, SAR China; 5 Graduate School of Engineering, Osaka University, Suita, 565-0871 Osaka Japan

## Abstract

High-entropy alloys (HEAs) are well known for their excellent high-temperature stability, mechanical properties, and promising resistance against oxidation and corrosion. However, their low-temperature applications are rarely studied, particularly in electronic packaging. In this study, the interfacial reaction between a Sn-3.0Ag-0.5Cu solder and FeCoNiCrCu_0.5_ HEA substrate was investigated. (Cu_0.76_, Ni_0.24_)_6_Sn_5_ intermetallic compound was formed the substrate at the interface between the solder and the FeCoNiCrCu_0.5_ HEA substrate. The average Sn grain size on the HEA substrate was 246 μm, which was considerably larger than that on a pure Cu substrate. The effect of the substrate on Sn grain size is due to the free energy required for the heterogeneous nucleation of Sn on the FeCoNiCrCu_0.5_ substrate.

## Introduction

High-entropy alloys (HEAs) have received increasing attention in the field of alloy research, and they have been extensively investigated^1–7^. These alloys are broadly defined as solid solution alloys that contain more than five principal elements in equal or approximately equal atomic percentages (at%). The atomic faction of each component is typically larger than 5 at%. HEAs exhibit excellent high-temperature stability, mechanical properties, and resistance against oxidation and corrosion^7–12^. In particular, FeCoNiCrCu_0.5_ with a face centered structure shows excellent high-temperature phase stability and steady hardness after various heat treatments^12^. Even though the excellent properties of FeCoNiCrCu_0.5_ have been proved in high-temperature conditions (300–800 °C)^9–15^, low-temperature applications have been rarely studied, particularly in electronic packaging.

Sn-rich solder on a Cu or Ni substrate is commonly utilized for interconnections in lead-free surface-mount technology^16,17^ and flip-chip solder joints^18–20^ owing to its low melting point (212 °C) and suitable thermal, mechanical, and wetting properties. Moreover, the microstructure and thickness of intermetallic compounds (IMCs) at an interface are important for the reliability of Sn-rich solder joints^16,21–23^. In recent studies, it was reported that coefficient of thermal expansion (CTE) mismatch between Sn grains caused crack propagation on grain boundaries^24^. Furthermore, Sn grain boundary, which was a channel for atomic diffusions, caused under-bump-metallization dissolution and serious major IMC formation in solder joints during electromigration^25^. Thus, the grain size distribution in a solder matrix on any substrate is critical. However, the investigation for Sn-rich solder soldered to HEA substrate is rare in literature, particularly for the effect of HEA substrate on Sn grain size and microstructure.

In this study, we demonstrate that Sn-3.0Ag-0.5Cu (SAC, wt%) alloy can be soldered to an FeCoNiCrCu_0.5_ HEA substrate (at%). The contact angles, the identification of IMCs at the interface, and the Sn grain size in the solder material are analyzed. Subsequently, an SAC solder on a Cu substrate (SAC-Cu) serves as a benchmark.

## Materials and Methods

**Figure 1:**
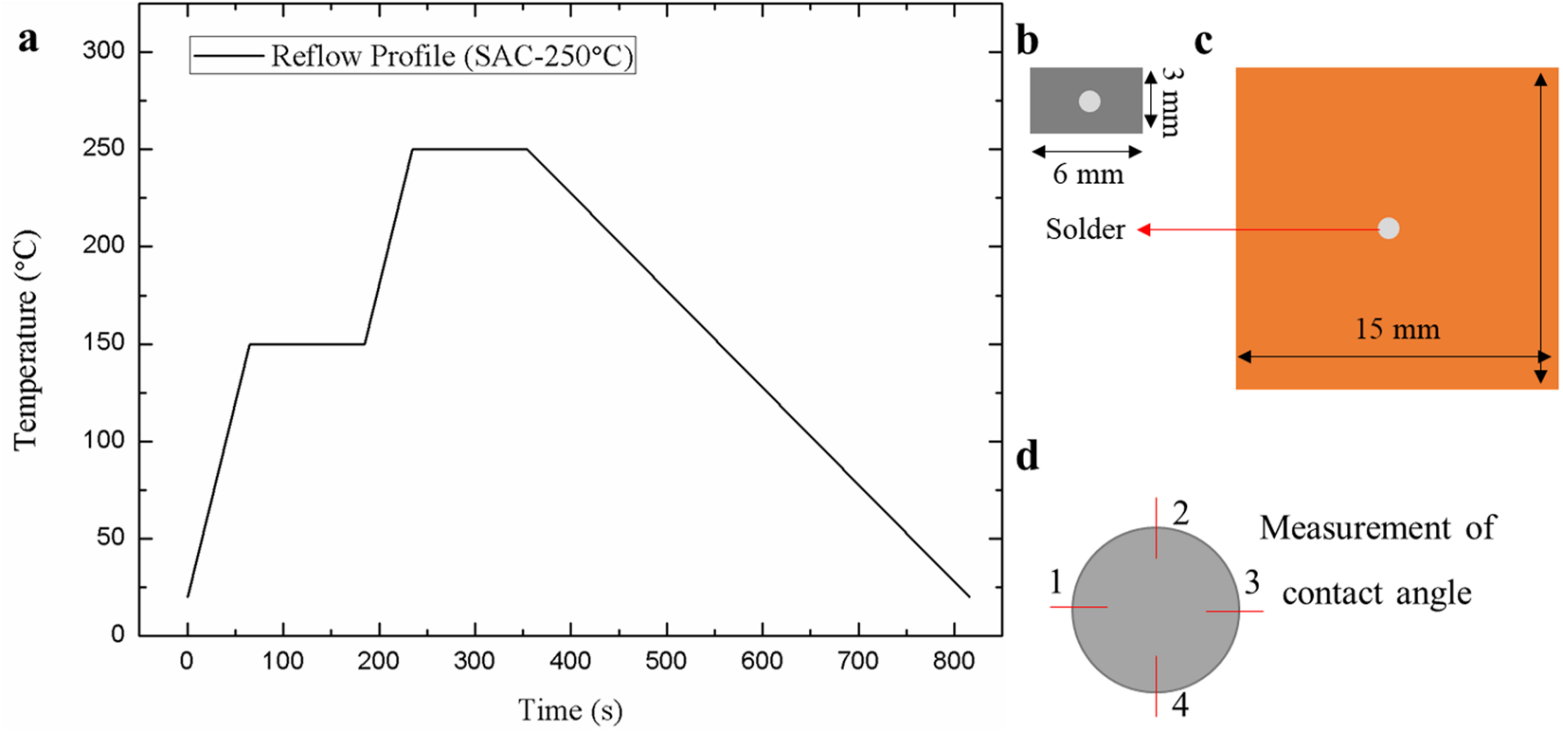
Experimental set-up. (**a**) Reflow profile using for reflow process. Sn-3.0Ag-0.5Cu solder on (**b**). FeCoNiCrCu0.5 substrates and (**c**). Cu substrates. (**d**) A schematic image of measured point of contact angles.

Figure 1a depicts a reflow profile for the fabrication of SAC-HEA and SAC-Cu samples, as shown in Fig. 1b,c, respectively. HEA substrates with a size of 3 mm × 6 mm × 3 mm were cut from HEA bulk remelted in an arc furnace with argon atmosphere. The fabrication and analysis of the FeCoNiCrCu_0.5_ HEA are presented in the Supplementary Information. Cu substrates with a size of 16 mm × 16 mm × 0.5 mm were commercially fabricated, and spherical SAC solders with a diameter of 0.76 mm were used.

After the reflow processes, the contact angles of the solders on the substrates in Fig. 1b,c were measured using 3D laser microscopes (KEYENCE, Japan). Each sample was measured at four points, as shown in Fig. 1d, and then, average contact angles were calculated. Backscattered electron images (BEIs) were recorded using a scanning electron microscope (JOEL-7100, Japan) to observe the thicknesses of the IMCs at the interface between the solder and substrate. Energy dispersive X-ray spectrometer (EDS, JOEL, Japan) was used to identify the IMCs. Sn grain size and orientation were analyzed through electron backscatter diffraction (EBSD, OIM Analysis, Japan). The data treatments for EBSD are provided by TSL OIM (OIM Analysis, Japan).

## Results and Discussion

**Figure 2:**
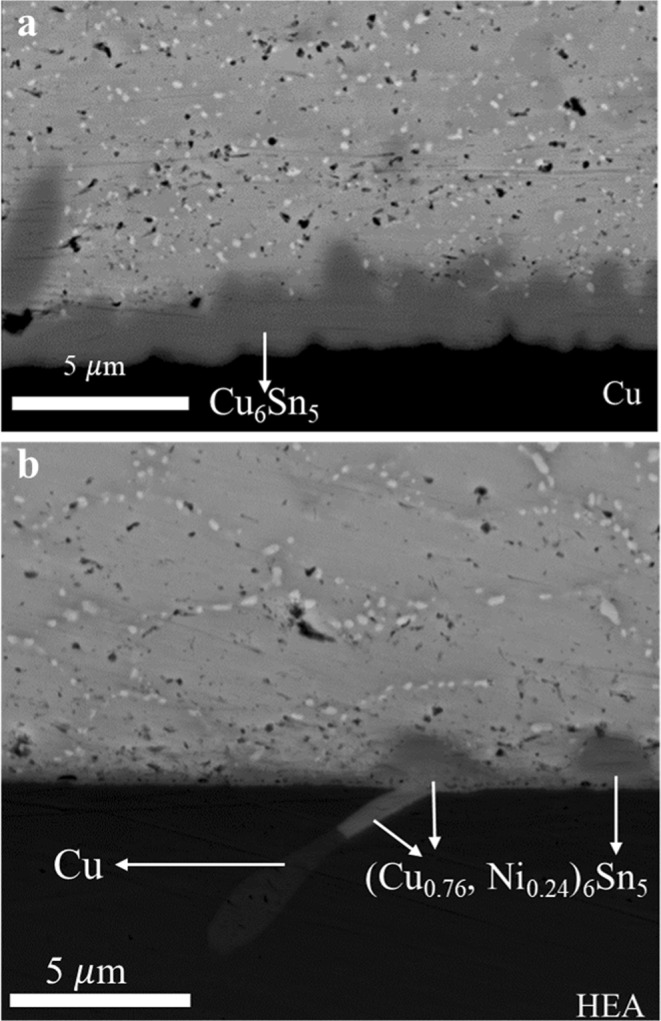
Cross-section backscattered electron images. (**a**) A backscattered electron image in a SAC solder with a layer of Cu_6_Sn_5_ intermetallic compound on a Cu substrate. (**b**) A backscattered electron image in a SAC solder with (Cu_0.76_, Ni_0.24_)_6_Sn_5_ intermetallic compound on a FeCoNiCrCu_0.5_ substrate.

Figure 2 shows the BEIs of the interface in the SAC-Cu and SAC-HEA samples. The composition of the IMCs at the interface is crucial for determining the major elements for soldering. Concerning the Cu substrate, numerous studies have reported that Cu_6_Sn_5_ is the main IMC for SAC/Cu systems in a reflow condition^16,17^. We confirm this fact in this study, as shown in Fig. 2a. In contrast, Fig. 2b shows that the IMC in SAC-HEA is (Cu_0.76_, Ni_0.24_)_6_Sn_5_ with a significantly small thickness identified by EDS. As IMC formation occurs not only at the interface between the solder and the Cu phase of the HEA but also at the interface between the solder and the Cu-free phase of the HEA, the Cu atoms for the IMC formation must partially come from the SAC solder. (Cu_1-y_, N_y_)_6_Sn_5_ IMC has been observed on Ni substrate in several studies^16,26^. This IMC is based on the Cu_6_Sn_5_ crystal structure with a Cu concentration of over 0.4% in solders^26^. In Ni-Cu-Sn system, time is not sufficient for the ternary compound Ni_26_Cu_29_Sn_45_ to form during the reflow process. Thus, Cu_6_Sn_5_ with some solution of Ni is required according to the thermodynamic calculation^16^. In this study, SAC on the HEA substrate with 25 at% Ni is extremely similar to SAC on a Ni substrate. Although it exhibits a relatively higher Cu and lower Ni at%, the formation of (Cu_1-y_, Ni_y_)_6_Sn_5_ IMC can be expected upon solidification at the interface. Furthermore, the partial consumption of the Cu atoms of the HEA substrate by the IMC formation is clearly observed. The limitation of reacting elements with Sn in the HEA is highly practical for decreasing IMC formation because the IMC is so brittle that it causes fracture. However, the IMC layer is discontinuous, which might affect joining reliability. Therefore, the process for the formation of a continuous layer of IMCs at the interface is an important goal in future.

**Figure 3:**
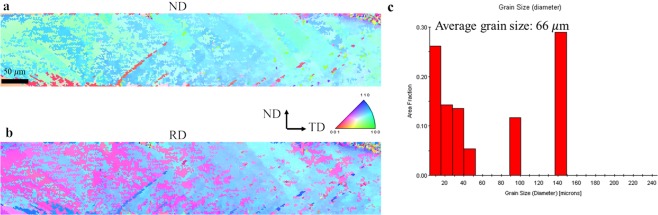
Analysis of electron back scatter diffraction by Sn inverse pole figure in a Sn-3.0Ag-0.5Cu solder on Cu substrate. EBSD orientation image maps in (**a**). Normal direction and (**b**). Rolling direction, respectively. (**c**) A distributions of Sn grain size.

**Figure 4:**
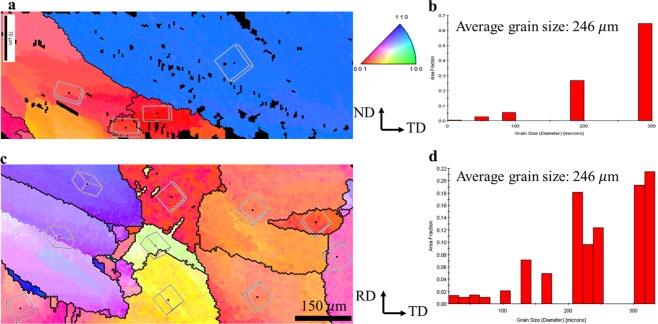
Analysis of electron back scatter diffraction by Sn inverse pole figure in Sn-3.0Ag-0.5Cu solders on FeCoNiCrCu_0.5_ substrates. (**a**) An EBSD orientation image map in the normal direction of a cross-section SAC solder with (**b**). Its distribution of Sn grain size. (**c**) An EBSD orientation image map in the normal direction of a planar SAC solder with (**d**) its distribution of Sn grain size.

Figure 3a depicts the cross-sectional EBSD orientation image map (OIM) by Sn inverse pole figure (IPF) in normal direction (ND, the direction vertical to the substrate) in an SAC solder on the Cu substrate. The Sn grain orientations are mostly close to [110] in ND, with significantly lower diffusivity of Cu in Sn compared to [001]^22^. The OIM in rolling direction (out of the plane direction) shown in Fig. 3b presents the distribution of the grains. Obviously, there is a large Sn grain with a grain size of approximately 145 μm and with small grains in the SAC-Cu sample. Small Sn grains distributed in large grains in SAC solders on Cu substrates have been presented in literature^27,28^, which are similar to the results of this study. In other words, this kind of microstructure is commonly observed in SAC-Cu samples. The average grain size is approximately 66 μm, as shown in the grain-size distribution in Fig. 3c. Figure 4a illustrates the cross-section EBSD OIM of an SAC-HEA sample by Sn IPF in ND. There are two large Sn grains with sizes of 290 μm and 186 μm, as shown in Fig. 4b. The grain orientations in ND are close to [110], with lower Cu diffusivity in Sn, and [001], with higher Cu diffusivity in Sn. For counting considerably more grains, Fig. 4c depicts the planar EBSD OIM of another SAC-HEA sample in ND. There are five grains with grain sizes larger than 200 μm, as shown in Fig. 4d. Note that according to Fig. 4a,c, the Sn grain orientation in SAC-HEA is random.

**Figure 5:**
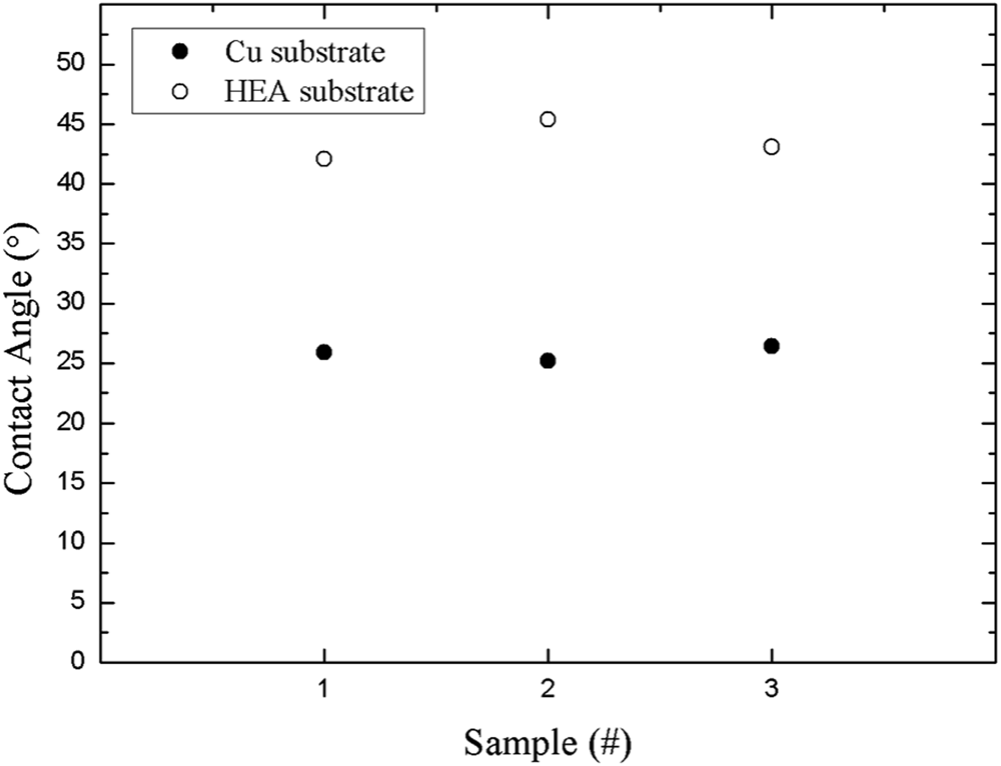
Distribution of contact angles of Sn-3.0Ag-0.5Cu solders on Cu and FeCoNiCrCu_0.5_ substrates.

**Figure 6:**
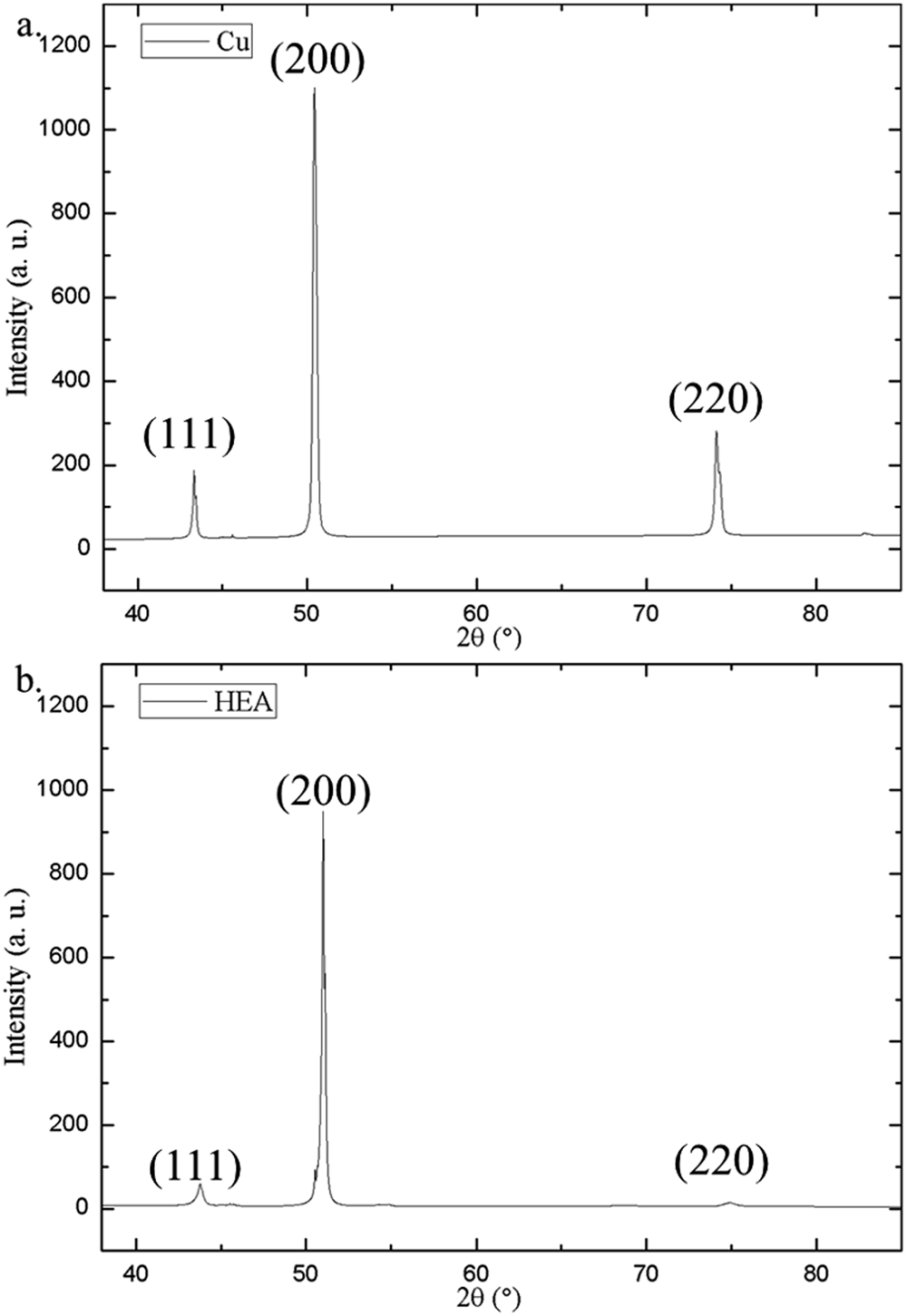
XRD pattern of substrates. (**a**) Cu. (**b**) HEA.

Nevertheless, the Sn grain size in SAC-HEA is remarkably larger than that in SAC-Cu. The effect of substrates on grain size is caused by the difference in the free energies required for heterogeneous nucleation in different substrates. The free energy required for heterogeneous nucleation (ΔG_*het*_) is defined by the following equation^29^:

1 $${{\rm{\Delta }}{\rm{G}}}_{het}={{\rm{\Delta }}{\rm{G}}}_{hom}\cdot {\rm{f}}({\rm{\theta }})$$

where ΔG_*hom*_ is the free energy required for homogeneous nucleation, and ${\rm{f}}({\rm{\theta }})$ can be expressed as

2 $${\rm{f}}({\rm{\theta }})=\frac{2-3\,\cos \,\theta +co{s}^{3}\theta }{4}$$

where ${\rm{\theta }}$ is the contact angle. Figure 5 shows the average contact angles of the SAC-Cu and SAC-HEA samples. The wettability of SAC-Cu is better than that of SAC-HEA. The average contact angles of the three SAC-Cu samples and three SAC-HEA samples are 25.2° and 43.5°, respectively. According to Equation (2), the values of f (${\rm{\theta }}$) for SAC-HEA and SAC-Cu are 0.051 and 0.0064, respectively. The two systems differ by a factor of 10. As f(${\rm{\theta }}$) is smaller, a lower ΔG_*het*_ induces frequent heterogeneous nucleation in a crystal on a solid surface. Therefore, the grain size of SAC-HEA, with higher ΔG_*het*_, is significantly larger than that of SAC-Cu, with lower ΔG_*het*_. Moreover, the distribution of grain orientation in the Cu substrate is analyzed through X-ray diffraction, and it is found to be random (Fig. 6a). The grain orientation in the HEA is also random and similar to literature (Fig. 6b)^12^. Thus, the effect of the microstructures of the substrates on the contact angles is negligible in this study. Interganular fracture is a common failure mode for metals. Therefore, an SAC solder on an HEA with few grain boundaries is a promising system that can reduce the probability of crack propagation along grain boundaries.

## Conclusion

In this study, based on large Sn grain sizes, we successfully spread SAC solder soldered on FeCoNiCrCu_0.5_ HEA substrate which exhibits excellent high-temperature stability and resistance against corrosion. The average Sn grain size in the solder was 246 μm, which was considerably larger than the value of 66 μm on the Cu substrate. (Cu_0.76_, Ni_0.24_)_6_Sn_5_ IMC was observed at the interface between the SAC solder and HEA. The effect of the substrate on Sn grain size was caused by the different free energies of the substrates required for heterogeneous nucleation. Larger Sn grains were formed in the SAC solder on the HEA substrate compared with that on the Cu substrate owing to higher free energy required for heterogeneous nucleation.

## Supplementary information

Supplementary information of Effect of FeCoNiCrCu0.5 High-entropy-alloy Substrate on Sn Grain Size in Sn-3.0Ag-0.5Cu Solder
