# Author Correction: Effect of FeCoNiCrCu_0.5_ High-entropy-alloy Substrate on Sn Grain Size in Sn-3.0Ag-0.5Cu Solder

**DOI:** 10.1038/s41598-020-67126-y

**Published:** 2020-07-22

**Authors:** Yu-An Shen, Chun-Ming Lin, Jiahui Li, Siliang He, Hiroshi Nishikawa

**Affiliations:** 1 Joining and Welding Research Institute (JWRI), Osaka University, Ibaraki, 567-0047 Osaka Japan; 2 Department of Mechanical Engineering, Minghsin University of Science and Technology, Hsinchu, 30401 Taiwan; 3 Department of Aviation Mechanical Engineering, China University of Science and Technology, Hsinchu, 312 Taiwan; 4 Department of electronic engineering, City University of Hong Kong, Hong Kong, SAR China; 5 Graduate School of engineering, Osaka University, Suita, 565-0871 Osaka Japan

Correction to: *Scientific Reports* 10.1038/s41598-019-40268-4, published online 06 March 2019

In the Supplementary Information file originally published with this Article, some text was inappropriately used and cited. These errors have been corrected in the Supplementary Information that now accompanies the Article.

