# Supplementary material for: Effect of FeCoNiCrCu0.5 High-entropy-alloy Substrate on Sn Grain Size in Sn-3.0Ag-0.5Cu Solder
Source: Sci Rep. 2019 Mar 6;9:3658. doi: 10.1038/s41598-019-40268-4 (PMC6403290; doi:10.1038/s41598-019-40268-4)
Supplement: Supplementary file 1 — Supplementary information of Effect of FeCoNiCrCu0.5 High-entropy-alloy Substrate on Sn Grain Size in Sn-3.0Ag-0.5Cu Solder [file 41598_2019_40268_MOESM1_ESM.docx]

**Supplementary information of**

**Effect of FeCoNiCrCu0.5 High-entropy-alloy Substrate on Sn Grain Size in Sn-3.0Ag-0.5Cu Solder**

Yu-An Shen^1*^, Chun-Ming Lin^2,3^, Jiahui Li^4^, Siliang He ^5^, Hiroshi Nishikawa^6*^,

^1^Jointing and Welding Research Institute (JWRI), Osaka University, Osaka, 5600047, Japan

^2^ School of Mechanical Engineering, Hefei University of Technology,

Hefei, 230009, China

^3^Department of Aviation Mechanical Engineering, China University of Science and Technology, Hsinchu, 312,Taiwan

^4^Department of Electronic Engineering, City University of Hong Kong, Hong Kong SAR, China

^5^Graduate School of Engineering, Osaka University, Osaka, Japan

* Corresponding author. Tel.: +81 668798685;

E-mail address: [r123845986@hotmail.com](mailto:r123845986@hotmail.com) (YA. Shen).

E-mail address: [nisikawa@jwri.osaka-u.ac.jp](mailto:nisikawa@jwri.osaka-u.ac.jp) (H. Nishikawa).

1. FeCoNiCrCu0.5 high-entropy alloy

The ingot for FeCoNiCrCu0.5 HEA^1^ was melted under an argon atmosphere in an arc furnace with a mixture of appropriate amounts of highly purity elements (99.99%), the Ingots were obtained in a copper mould. Each sample was reversed and re-melted four times in order to assure chemical homogeneity. The final samples were button-shaped, approximately 8 mm thick, with a shiny surface. The microstructure and chemical composition of the alloys were analyzed by scanning electron microscope (SEM, JEOL JEOL JSM-5410) and energy dispersive spectrometer (EDS).

2. Measurement of Contact Angle

The contact angle is the angle, conventionally measured through the liquid, where a liquid–vapor interface meets a solid surface. It quantifies the wettability of a solid surface by a liquid via the Young equation. A given system of solid, liquid, and vapor at a given temperature and pressure has a unique equilibrium contact angle. However, in practice contact angle hysteresis is observed, ranging from the so-called advancing (maximal) contact angle to the receding (minimal) contact angle. The equilibrium contact is within those values, and can be calculated from them. The equilibrium contact angle reflects the relative strength of the liquid, solid, and vapor molecular interaction.

[
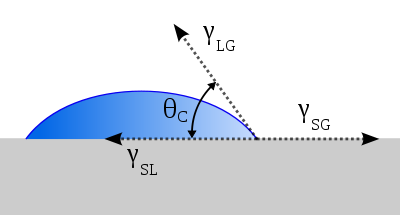
](https://en.wikipedia.org/wiki/File:Contact_angle.svg)

Schematic of a liquid drop showing the quantities in the Young equation.

The shape of a liquid–vapor interface is determined by the [Young–Laplace equation](https://en.wikipedia.org/wiki/Young%E2%80%93Laplace_equation), with the contact angle playing the role of a [boundary condition](https://en.wikipedia.org/wiki/Boundary_value_problem) via the [Young equation](https://en.wikipedia.org/wiki/Wetting#Simplification_to_planar_geometry.2C_Young.27s_relation).


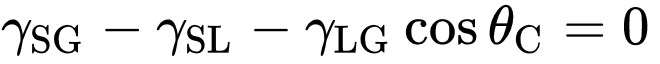
The theoretical description of contact arises from the consideration of a [thermodynamic](https://en.wikipedia.org/wiki/Thermodynamics) [equilibrium](https://en.wikipedia.org/wiki/Thermodynamic_equilibrium) between the three [phases](https://en.wikipedia.org/wiki/Phase_(matter)): the [liquid](https://en.wikipedia.org/wiki/Liquid) phase (L), the [solid](https://en.wikipedia.org/wiki/Solid) phase (S), and the gas or [vapor](https://en.wikipedia.org/wiki/Vapor) phase (G) (which could be a mixture of ambient atmosphere and an equilibrium concentration of the liquid vapor). (The "gaseous" phase could be replaced by another [immiscible](https://en.wikipedia.org/wiki/Miscibility) liquid phase.) If the solid–vapor [interfacial energy](https://en.wikipedia.org/wiki/Surface_energy) is denoted by γ S G , the solid–liquid interfacial energy by γ S Land the liquid–vapor interfacial energy (i.e. the [surface tension](https://en.wikipedia.org/wiki/Surface_tension)) by γ L G then the equilibrium contact angle θ Cis determined from these quantities by the [Young equation](https://en.wikipedia.org/wiki/Wetting#Simplification_to_planar_geometry.2C_Young.27s_relation):


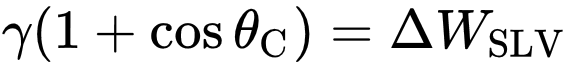
The contact angle can also be related to the work of [adhesion](https://en.wikipedia.org/wiki/Adhesion) via the [Young–Dupré equation](https://en.wikipedia.org/wiki/Young%E2%80%93Dupr%C3%A9_equation):

where Δ W S L Vis the solid – liquid adhesion energy per unit area when in the medium V

3D Laser microscopes are designed to generate high-resolution optical images as well as surface shape characterization quickly and accurately. Laser microscopes combine the advantages of magnified observation devices and measurement devices in order to obtain both full-focus images and reliable 3D shape analysis. With these devices, there are no size, shape, or material limitations. Observations can be made at room temperature without the need for a vacuum, and operations are easy to perform in a manner similar to optical microscopes. There is no need for pre-processing of samples and it is possible to perform color observation. All of these factors enable quick and accurate analysis of samples. These microscopes can also be used to observe the surface layer, inside, and bottom layer of transparent targets and to measure film thickness.

**Reference:**

1. Lin, C. M. & Tsai, H. L. Equilibrium phase of high-entropy FeCoNiCrCu0.5 alloy at elevated temperature. *J. Alloys Compd.* **489**, 30-35 (2010).
